# BAP31, a promising target for the immunotherapy of malignant melanomas

**DOI:** 10.1186/s13046-015-0153-6

**Published:** 2015-04-18

**Authors:** Shaojuan Yu, Fuli Wang, Li Fan, Yuying Wei, Haitao Li, Yuanjie Sun, Angang Yang, Boquan Jin, Chaojun Song, Kun Yang

**Affiliations:** Department of Immunology, Fourth Military Medical University, 169 Changle West Road, Xi’an, 710032 People Republic of China; Department of Cardiology, First Hospital of Xi,an, 30 Fenxiang, Xi’an, 710003 People Republic of China; Department of Urology, Xijing Hospital, 125 Changle West Road, Xi’an, 710032 People Republic of China; Department of Pharmaceutical Analysis, Fourth Military Medical University, 169 Changle West Road, Xi’an, 710032 People Republic of China

**Keywords:** Malignant melanomas, Cancer immunotherapy, BAP31, DNA vaccine, LAMP

## Abstract

**Purpose:**

Malignant melanoma’s (MM) incidence is rising faster than that of any other cancer in the US and the overall survival at 5 years is less than 10%. B cell associated protein 31 (BAP31) is overexpressed in most MMs and might be a promising target for immunotherapy of this disease.

**Experimental design:**

Firstly, we investigated the expression profiles of human BAP31 (hBAP31) and mouse BAP31 (mBAP31) in human and mouse normal tissues, respectively. The expression level of hBAP31 in human MMs and mBAP31 in B16 melanoma cells was also analyzed. Then we constructed novel mBAP31 DNA vaccines and tested there ability to stimulate mBAP31-specific immune responses and antitumor immunity in B16 melanoma-bearing mice.

**Results:**

For the first time, we found that protein expression of hBAP31 were dramatically upregulated in human MMs when compared with human normal tissues. Predominant protein expression of mBAP31 was found in mouse B16 melanoma cells but not in mouse important organs. When mice were immunized with mBAP31 DNA vaccines, strong cellular response to mBAP31 was observed in the vaccinated mice. CTLs isolated from immunized mice could effectively kill mBAP31-positive target mouse B16 melanoma tumor cells *in vitro* and vaccination with mBAP31 DNA vaccines had potent anti-tumor activity in therapeutic model using B16 melanoma cells.

**Conclusions:**

These are the first data supporting a vaccine targeting BAP31 that is capable of inducing effective immunity against BAP31-expressing MMs and will be applicable to human MMs and hBAP31 DNA vaccine warrants investigation in human clinical trials.

**Electronic supplementary material:**

The online version of this article (doi:10.1186/s13046-015-0153-6) contains supplementary material, which is available to authorized users.

## Introduction

Cancer malignancies are among the most life-threatening diseases worldwide [1]. MM consists approximately 3% of all cancers and its incidence continues to rise. Although treated by adequate surgery, the overall survival of MM at 5 years is less than 10%. For disseminated MM, the appropriate systemic medical treatment is still controversial [2,3]. Identification of new tumor antigens expressed in melanoma and progresses in the molecular biology and immunotherapy should in the near future translate into molecular-based therapeutic strategies [4]. Followed the discovery of a lot of tumor antigens and development of novel strategies, cancer immunotherapy has significantly developed during the last decades and completes the therapeutic arsenal [5,6]. Human preclinical trials of immunotherapy based on the newly defined tumor antigens highly expressed by MMs have now been reported and cast new light on the therapeutic strategies of the disease [7,8].

BAP31 is an ER chaperon that associates with newly synthesized integral membrane proteins and controls their fate. BAP31 is an integral ER-resident membrane protein and a component of several large protein complexes [9]. In addition to its role as ER chaperon, BAP31 plays an important role in apoptosis. BAP31 could act as a regulator of procaspase-8 L processing [10] and could be cleaved by caspase-8 [11,12]. The cleaved BAP31 fragment, p20, is a potent inducer of apoptosis when expressed ectopically. Prominent BAP31 protein expression is restricted to a minority of cells in normal human tissues although its RNA transcripts are ubiquitously expressed [13,14].

For the first time, we found that protein expression of hBAP31 were dramatically upregulated in human MM tissues when compared with human normal tissues, with a total positive rate of 86.5% (122 of 141 cases). And predominant protein expression of mBAP31 was found in mouse B16 melanoma cells but not in mouse important organs. So we hypothesized that BAP31 might be used as a promising immunotherapy target for MMs. In this study we constructed mBAP31 DNA vaccines and lysosome-associated membrane protein (LAMP) was used to enhance the immune response against mBAP31. LAMP can target and bind the endosome/lysosome through the LAMP transmembrane/cytoplasmic domain. The luminal domain of LAMP is then integrated into the lysosome. Consequently, one strategy commonly is to insert the target DNA into LAMP as a LAMP/antigen chimera and could greatly enhance the immune response against a number of antigens [15,16]. When C57BL/6 mice were immunized with LAMP-mBAP31 chimera DNA vaccine or mBAP31 DNA vaccine, strong cellular response to mBAP31 was observed and no autoimmune disorders were observed in the vaccinated mice. Moreover, CTLs isolated from mBAP31 DNA vaccine immunized C57BL/6 mice could effectively kill mBAP31-positive target mouse B16 melanoma tumor cells *in vitro*. Furthermore, in a therapeutic model, vaccination with mBAP31 DNA vaccine had potent anti-tumor activity even in the established B16 melanoma. These are the first data supporting a vaccine targeting BAP31 that will be applicable to human MMs.

## Materials and methods

### Mice and cell lines

All animal studies were conducted under a protocol approved by Animal Care Research Advisory Committee of Fourth Military Medical University and all experiments involving mice were according to the Guidelines of the Animal Research Ethics Board of Fourth Military Medical University. All experiments involving mice were performed under fully anesthetized by inhalation of a mixture of oxygen with 5% isoflurane, and all efforts were made to minimize suffering.

Female C57BL/6 (H-2^b^) mice (8 weeks old) were maintained and treated in accordance with recommendations for the proper use and care of laboratory animals. 293 T, mouse colon carcinoma cell line CT26 (H-2^d^) and mouse melanoma cell line B16 (H-2^b^) were purchased from American Type Culture Collection (ATCC) and cultured according to standard guidelines.

### IHC staining

Sections were firstly dewaxed and hydrated. Endogenous peroxidase activity was blocked with methanol containing 3% H_2_O_2_ for 10 min, and nonspecific binding was blocked by normal goat serum (10%) for 10 min. The sections were then dipped into mAbs specific for human or mouse BAP31 developed in our lab. Normal mouse IgG was applied as negative control. After three washes in PBS, the slices were dipped into biotin-conjugated goat anti-mouse IgG (promega) for 1 h at room temperature followed by streptavidin-HRP complex incubation for another 1 h. Then antibody complexes were visualized by incubation with DAB chromogen. Sections were counterstained with Mayer’s hematoxylin for 30 s, dehydrated through gradient ethanol, cleared in dimethyl benzene, mounted, and examined using light microscopy.

### Small interference RNA

The mammalian expression vector, pSUPER.retro.circular.stuffer (OligoEngine), was used for expression of siRNA in B16 cells. The gene-specific insert specifies a 19-nucleotide sequence (ggtgaacctccagaacaat) corresponding to nucleotides 339–357 downstream of the transcription start site of mBAP31, which is separated by a 9-nucleotide non-complementary spacer (tctcttgaa) from the reverse complement of the same 19-nucleotide sequence. This vector was referred to as pSUPER-mBAP31. A control vector (pSUPER-Control) was constructed using a 19-nucleotide sequence (gcgcgctttgtaggattcg) with no significant homology to any mammalian gene sequence and therefore serves as a non-silencing control (OligoEngine). These sequences were inserted into the pSUPER.retro.circular.stuffer backbone.

B16 cells, maintained in RPMI 1640 containing 10% FCS, were plated onto 6-well plates at 2 × 10^5^ cells per well. After cultured in 37°C, 5%CO_2_ for 24 h, cells were transfected with 4 μg of RNAi plasmid hybrids using Lipofectamine 2000 reagent (Invitrogen) according to the manufacturer’s instructions. Stable transfected cell lines were screened and selected with 0.5 μg/ml puromycin (Sigma). Clonal cell lines were selected by Western blotting to confirm lack or low expression of mBAP31.

### Western blotting analysis

Cell lysates (20 μg) were separated by SDS-PAGE and transferred onto Immobilon-P membrane (Millipore, Billerica, MA). The membrane was then blocked with 5% skim milk in PBST (0.29% Na_2_HPO_4_, 0.8% NaCl, 0.02% KCl, and 0.05% Tween-20, pH7.4) for 1 h at room temperature (RT) and then incubated with anti-mBAP31 mAb (diluted to 5 μg/mL) or control mAb for 1 h at RT. After washing three times with PBST, the membrane was incubated with HRP-conjugated goat anti-mouse IgG (1:1500 v/v, Promega) for 1 h at RT. Finally, the membrane was developed using an ECL kit (GE Healthcare) and exposed to Agfa x-ray film. Anti-β-actin mAb was used as internal standard for all samples.

### Construction of DNA vaccine encoding mBAP31

The p43 and p43-LAMP vectors were kind gifts of Dr. August. Full length of mBAP31 was cloned, inserted into the vectors and termed p-mBAP31 and p-LAMP/mBAP31 respectively. The vaccination plasmids were produced by transforming *Escherichia coli* DH5a, and were then purified to remove endotoxin (Qiagen, Valencia, CA, USA). These plasmids were also constructed to carry enhanced green fluorescent protein (EGFP), p-mBAP31/EGFP and p-LAMP/mBAP31/EGFP, for expedient analysis of protein expression. EGFP-tagged protein expressions were studied by transfecting them into 293 T cell lines. EGFP expression was observed 24–48 h after transfection by fluorescence microscopy.

### Immunization and evaluation of DNA vaccine immune response

Three groups female C57BL/6 mice were immunized subcutaneously at the base of the tail with 50 μg of the specified endotoxin-free DNA plasmid (p-mBAP31, p-LAMP/mBAP31) diluted in phosphate-buffered saline (PBS) or PBS as negative control. The mice were boosted twice every 3 weeks with the same plasmid. Immunized mice were observed of clinical manifestation of autoimmune diseases such as weight loss, hair/skin disorders, diarrhea or neurological disorders. 2 weeks after the third DNA injection, the frequency of cells producing IFN-γ in splenocytes was measured by ELISPOT. In briefly, Single cell suspensions depleted of red blood cells were prepared from freshly isolated immunized mouse splenocytes and washed 2 times with RPMI 1640 and then resuspended in RPMI 1640 contained 10% FBS. BAP31 specific IFN-γ production was determined by a standard ELISPOT assay following a 24 h incubation of splenocytes (10^6^ per well) with synthetic overlapping mBAP31 peptides (1 μg/mL, 15-mer peptides spanning the mBAP31 protein, each overlapping the next by 9 amino acids). As a negative control, splenocytes cells were pulsed with the irrelevant 15-mer peptide of Hantaan virus (QTADWLSIIVYLTSF). ConA and recombinant mBAP31 (1 μg/mL) were used as positive controls.

### Cytotoxcity assay

Cytoxicity of CTLs of immunized mice was determined by quantitative measurements of the release of lactic dehydrogenase (LDH). The mouse melanoma B16 cells (H-2^b^), colon carcinoma CT26 cells (H-2^d^) or 2E8 cells (mBAP31-depleted B16 cells) were pulsed with or without corresponding peptides (2 μg/mL) and used as target cells. CD8^+^ cells, isolated from splenocytes of immunized C57BL/6 mice by magnetic beads conjugated with anti-CD8 mAbs (BD pharmingen), served as effector cells. CTL assays were performed with effector cells (E) and target cells (T) (1 × 10^4^ cells/well) mixed together at ratios of 50:1, 25:1 or 12.5:1 in a final volume of 100 μl. After 4 h incubation at 37°C, 50 μl of the cultured supernatants was collected to assess the amount of LDH release using Non-Radioactive Cytotoxicity Assay kits (Promega). The percentage of target cell lysis was calculated from the following equation: 100 × (A-B)/(C-D), where A is the reading value of experimental signal, B is spontaneous background signal value of the effector cells, C is maximum signal value from target cells, and D is spontaneous background signal value of the target cells.

### Therapeutic anti-tumor model of mBAP31 DNA vaccine in C57BL/6 mouse

C57BL/6 mice were transplanted subcutaneously with 5 × 10^4^ B16 cells per mouse at day 0. On day 3, mice were randomized and divided into three groups (n = 8), immunized subcutaneously at the base of the tail with 50 μg of the DNA plasmid (p-mBAP31, p-LAMP/mBAP31) and PBS as negative control. Vaccination was repeated on days 10, 17, and 24 with the same plasmid. Tumor dimensions were measured serially, and tumor volumes were calculated using the following formula: long axis × (short axis)^2^ × 0.52 [17]. On day 42, B16 melanoma tumor bearing mice from each group were sacrificed and the tumors were removed from the mice.

### Statistical analysis

Differences of IFN-γ production in ELISPOT assays and tumor volumes in tumor therapeutic model in C57BL/6 mice were assessed using student *t* test. All *P* values are two tailed and all statistical analyses used SPSS 17.0 software. Differences at *P* < 0.05 were considered statistically significant.

## Results

### Protein expression profiles of hBAP31 in human MMs

**Figure 1:**
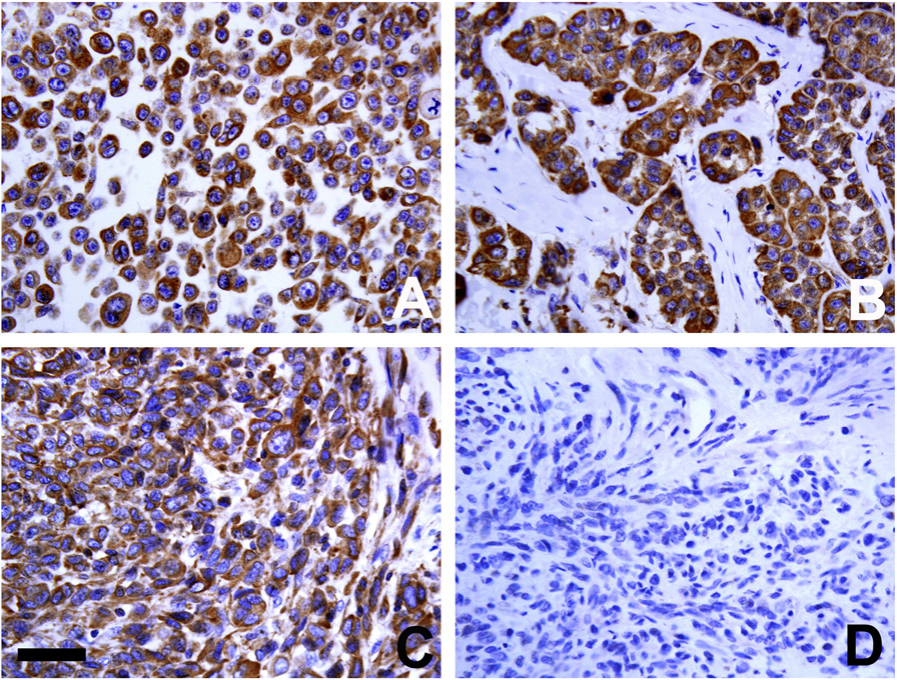
Immunoperoxidase staining of human BAP31 in human MMs. Representative expression patterns of BAP31 in human MMs **(A**-**C)**. The majority of tumor cells were intensely stained in the cytoplasm. Normal mouse IgG was applied as negative control **(D)**. Bar: 50 μm.

To assess the protein expression levels of hBAP31 in MMs, we employed IHC analysis using mAbs against hBAP31 established in our lab [18]. Of 141 MMs, 122 cases (86.5%) were hBAP31 positive. Representative expression patterns of hBAP31 in MMs were shown in Figure 1. The majority of tumor cells were intensely stained in the cytoplasm.

### Protein expression profiles of hBAP31 in human normal tissues

**Figure 2:**
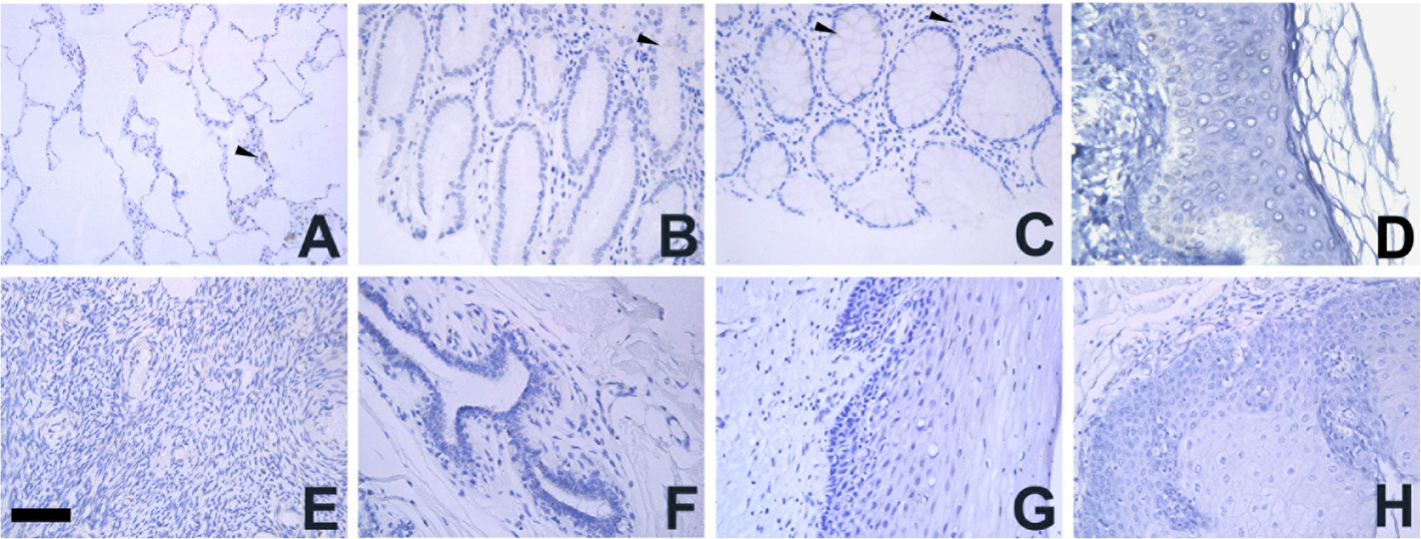
Expression profiles of human BAP31 in normal human tissues analyzed by IHC staining. There was marginal immunoreactivity for BAP31 in the lung **(A)**, colon **(B)** and rectum **(C)**. The brown products in these tissues were restricted to minority of cells. Whereas the skin **(D)** ovary **(E)**, breast **(F)**, cervix **(G)** and esophagus **(H)** were negative for BAP31. Bar: 50 μm.

Previous studies had shown that hBAP31 protein expression is restricted to a minority of cells in normal human tissues analyzed by immunohistochemistry [14]. The protein expression profiles of hBAP31 in human normal tissues in our study were consistent with previous report. In this experiment, all negative controls showed no specific positive reaction. Prominent hBAP31 protein expression analyzed by IHC staining in this study is restricted to a minority of cells in lung, colon and rectum. hBAP31 protein expression was negative in skin, ovary, breast, cervix and esophagus (Figure 2).

### Protein expression profiles of mBAP31 in mouse normal tissues

**Figure 3:**
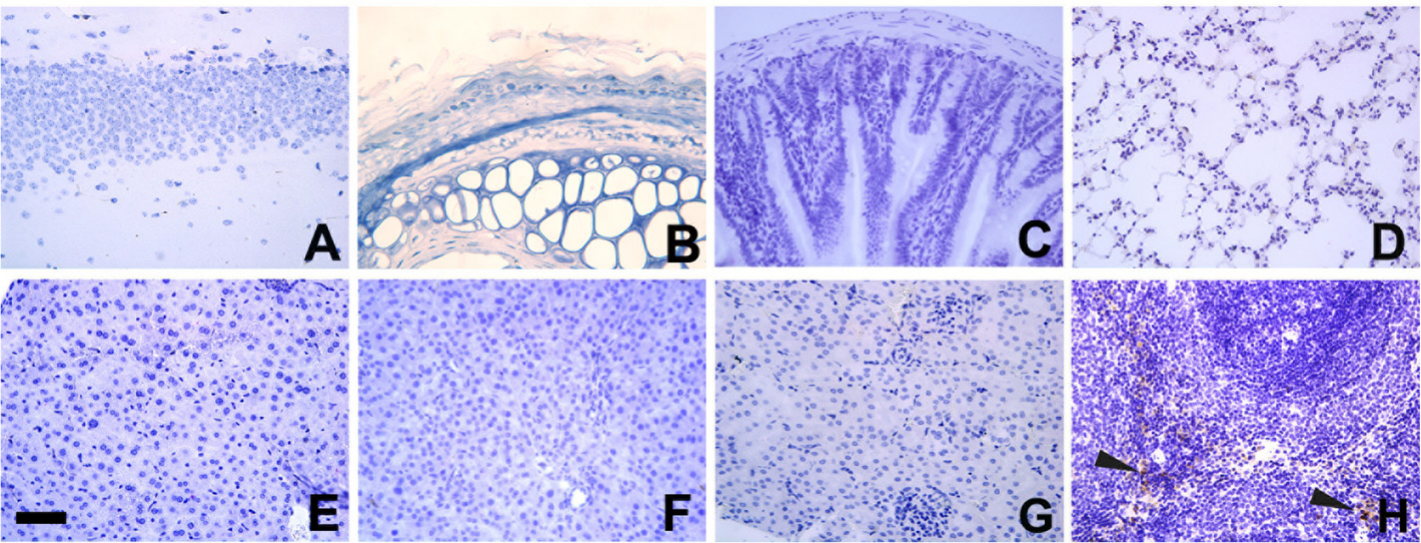
Expression profiles of mBAP31 in normal mouse tissues. We performed IHC staining to investigate the protein expression of mBAP31 in important organs of C57BL/6 mice. BAP31 reactivity was not found in mouse brain **(A)**, skin **(B)**, colon **(C)**, lung **(D)**, liver **(E)**, pancreas **(F)** or kidney **(G)**. Strong positive products were restricted to minority of mouse splenocytes **(H)**. Bar: 50 μm.

Although northern blotting analyses have revealed that mBAP31 RNA transcripts are ubiquitously expressed [13], there are no data on the mBAP31 protein expression profiles of mouse normal tissues. Using the mAbs against mBAP31 generated in our lab, we performed IHC staining to investigate the protein expression of mBAP31 in important organs of C57BL/6 mice. mBAP31 reactivity was not found in mouse brain, skin, colon, lung, liver, pancreas or kidney. Strong positive products were only found in minority of mouse splenocytes (Figure 3). The mechanism of the discrepancy of the expression level between the mRNA and protein of hBAP31 and mBAP31 should be investigated more intensely. Moreover, western blot analysis was performed to confirm the expression profiles of mBAP31 in mouse normal tissues (Additional file 1: Figure S1).

### Expression of mBAP31 protein in B16 cells, CT26 cells and siRNA B16 cells

Western blot analysis was performed to determine the expression profiles of mBAP31 protein in B16 cells, CT26 cells and siRNA B16 cells. As shown in Additional file 2: Figure S2, there is a special 28KD band in the film suggesting high protein expression of mBAP31 in B16 cells and CT26 cells. B16 cells were then transfected with the pSUPER-mBAP31 or pSUPER-Control, and selected with puromycin for 3 weeks. The pSUPER-BAP31 and pSUPER-Control cells were cloned and analyzed BAP31 expression by Western blot. One pSUPER-mBAP31-transfected clonal cell line named 2E8 with a remarkable reduction in mBAP31 protein expression was obtained after cultured for 2 months compared with the pSUPER-Control transfected clonal cell line 1H12 and non-transfected B16 cells. Anti-β-actin mAb was used as internal standard for all samples.

### Expression of mBAP31 protein in mBAP31 DNA vaccine transfected cells

The mBAP31 DNA vaccine was constructed with mouse homologue. The DNA vaccine must be able to express in mammalian cells for subsequent processing and presentation to T cells. To evaluate the expression levels of DNA vaccine vectors in mammalian cells, plasmids of EGFP-tagged mBAP31 and LAMP/mBAP31 were transfected into 293 T cells. The expression levels were analyzed by fluorescence microscopy and no significant difference in the fluorescence intensity of the transfected cells was observed, suggesting similar synthesis and translation rates for the two EGFP-tagged molecules (Additional file 3: Figure S3).

### Vaccination with mBAP31 DNA vaccine could elicit high level of cellular immune responses in C57BL/6 mice

**Figure 4:**
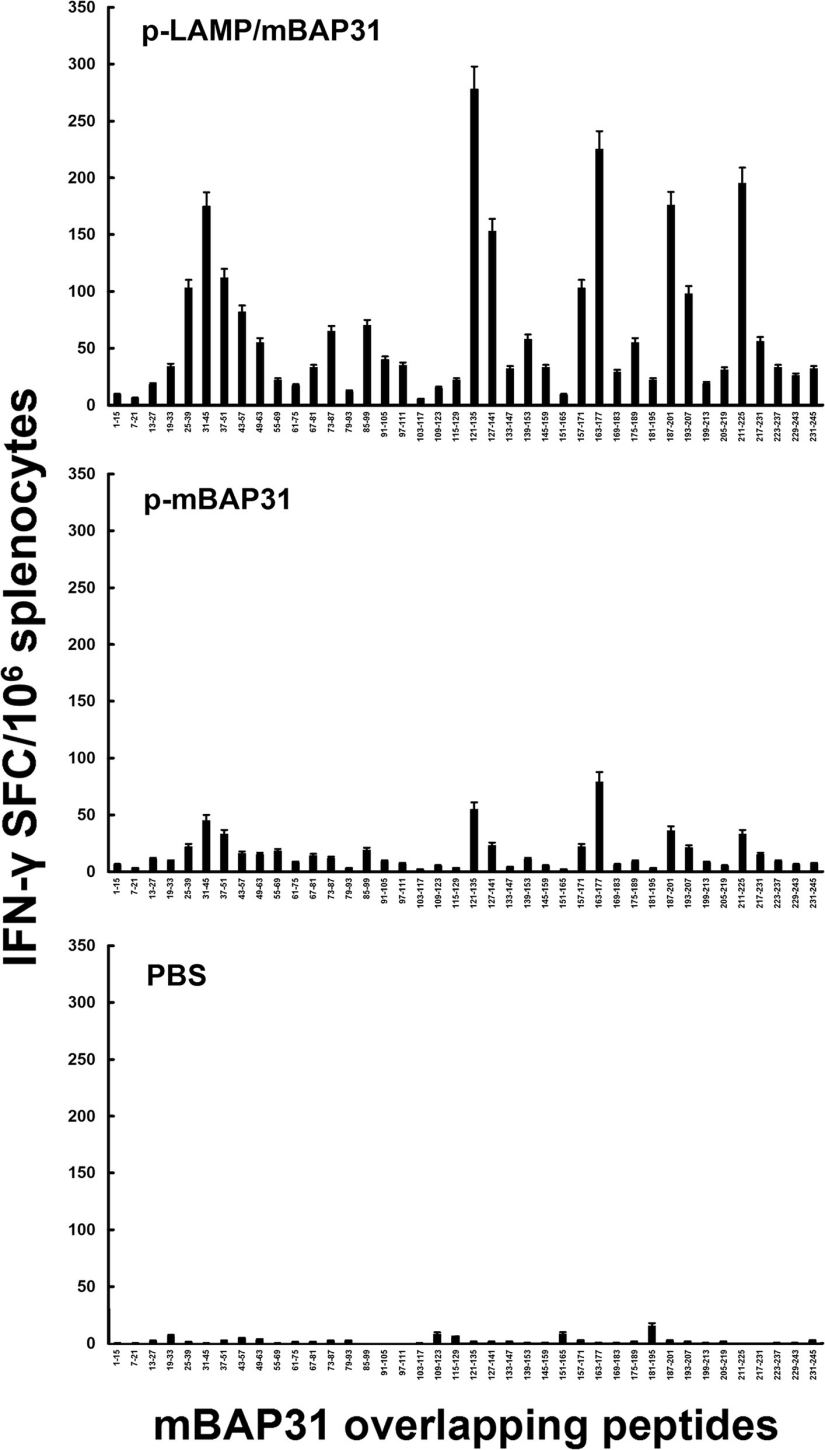
Induction of mBAP31-specific IFN-γ production by T cells following mBAP31 DNA vaccine vaccination in C57BL/6 mice. Three groups (n = 6) of female C57BL/6 mice were immunized subcutaneously at the base of the tail with the specified DNA plasmid (p-mBAP31, p-LAMP/mBAP31) or PBS and were boosted twice every 3 weeks. 2 weeks after the third vaccination, mice were sacrificed and the splenocytes were harvested. The frequency of cells producing IFN-γ in splenocytes was measured by ELISPOT. The number of IFN-γ-producing cells of p-LAMP/mBAP31 group was significantly higher than that of p-mBAP31 and control group.

Three groups of female C57BL/6 mice were immunized subcutaneously at the base of the tail with the specified DNA plasmid (p-mBAP31, p-LAMP/mBAP31, PBS as negative control) and were boosted twice every 3 weeks with the same plasmid. Immunized mice exhibited no clinical manifestation of autoimmune diseases such as weight loss, hair/skin disorders, diarrhea or neurological disorders. 2 weeks after the third DNA injection, the frequency of cells producing IFN-γ in splenocytes was measured by ELISPOT. The results of ELISPOT showed that T cell responses to mBAP31 were detected after three immunizations and the number of IFN-γ-producing cells of p-LAMP/mBAP31 group was significantly higher than that of p-mBAP31 and control group (Figure 4, *P* < 0.05), indicating that vaccination with mBAP31 DNA vaccine, especially LAMP chimera mBAP31 DNA vaccine, stimulated the expansion of mBAP31-specific T cells.

### Vaccination with mBAP31 DNA vaccine elicits mBAP31-specific cytotoxic T cells

**Figure 5:**
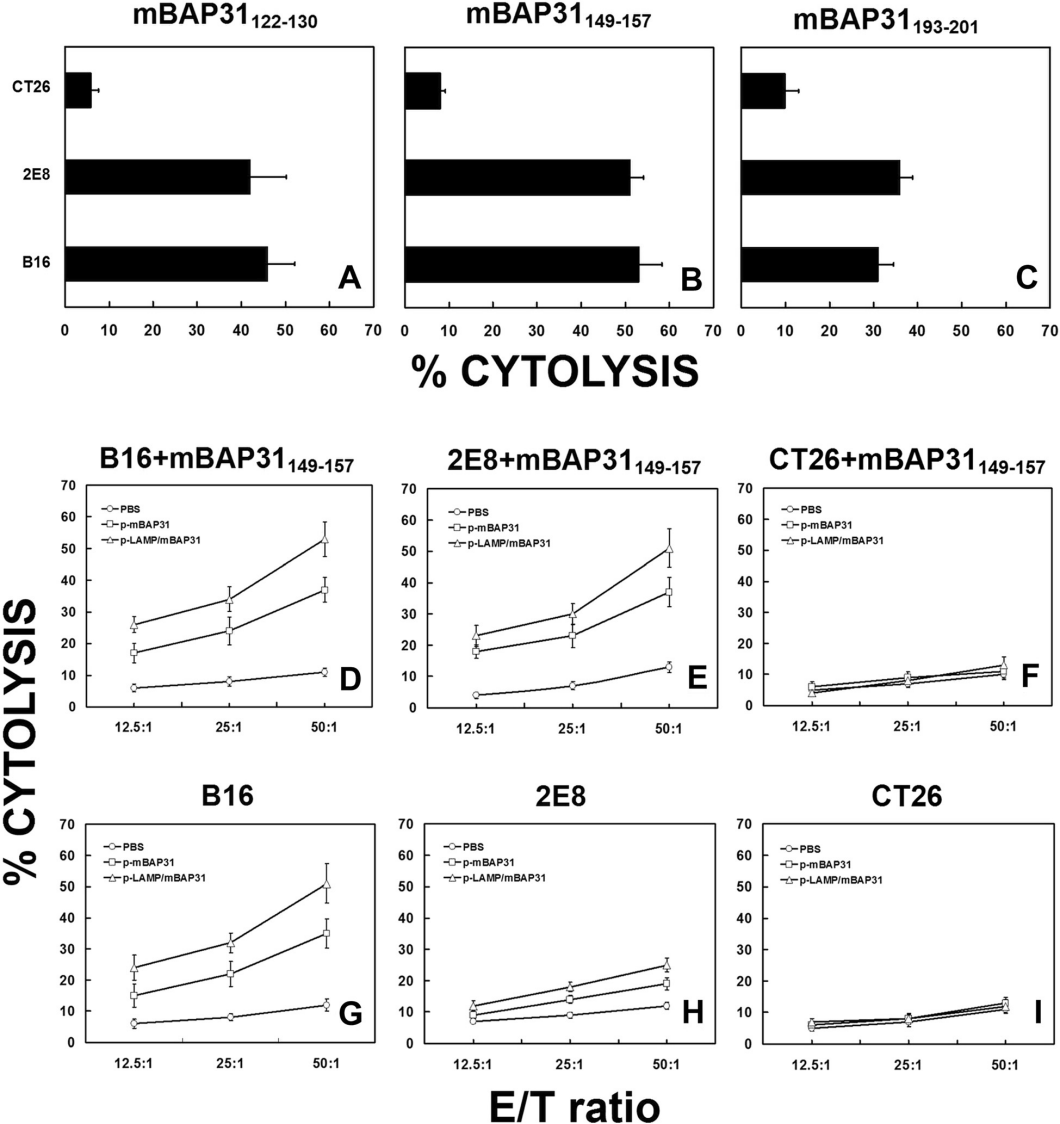
LDH release assay for cytotoxicity of CTLs from mBAP31 vaccinated mice against murine cancer cell lines. Cytotoxic activities of CTLs isolated from the splenocytes of the p-LAMP/mBAP31 vaccinated C57BL/6 mice (H-2^b^) against mBAP31 peptides pulsed mouse colon cancer cell lines CT26 (H-2^d^), 2E8 (mBAP31 depleted B16 cells, H-2^b^) and B16 (H-2^b^) were detected by LDH release assay at effector-to-target ratio of 50:1. Peptides used were indicated in abscissa **(A**-**C)**. Cytotoxic activities of CTLs from p-LAMP/mBAP31 (△), p-mBAP31 (□) or PBS (○) vaccinated C57BL/6 mice (H-2^b^) against B16 cells pulsed with mBAP31_149–157_ expressing both H-2^b^ and mBAP31 **(D)**, 2E8 cells (mBAP31 depleted B16 cells) pulsed with mBAP31_149–157_ expressing H-2^b^ but much lower mBAP31 than B16 cells **(E)**, CT26 cells pulsed with mBAP31_149–157_ expressing H-2^d^ and mBAP31 **(F)**, B16 cells expressing both H-2^b^ and mBAP31 **(G)**, 2E8 cells expressing H-2^b^ but much lower mBAP31 than B16 cells **(H)** and CT26 cells expressing H-2^d^ and mBAP31 **(I)**.

Cytoxicity of CTLs of immunized mice was determined by quantitative measurements of the release of LDH. The mouse melanoma B16 cells (H-2^b^), colon carcinoma CT26 cells (H-2^d^) or 2E8 cells (mBAP31-depleted B16 cells) were pulsed with or without corresponding peptides (2 μg/mL) and used as target cells. CD8^+^ cells were isolated from splenocytes of immunized C57BL/6 mice by magnetic beads conjugated with anti-CD8 mAbs and served as effector cells. The CD8^+^ cells from both p-mBAP31 and p-LAMP/mBAP31 immunized mice could efficiently kill B16 cells, mBAP31 peptides pulsed 2E8 cells but not 2E8 cells without pulsing mBAP31 peptides, CT26 cells (pulsed with or without mBAP31 peptides) at 50:1 ratio. And LAMP molecule could significantly enhance the cytotoxicity of mBAP31-specific CTLs against B16 cells (Figure 5). Thus, mBAP31 DNA vaccine could effectively induce anti-mBAP31 CTLs with anti-tumor activity.

### Vaccination with mBAP31 DNA vaccine suppresses tumor growth in a therapeutic model

**Figure 6:**
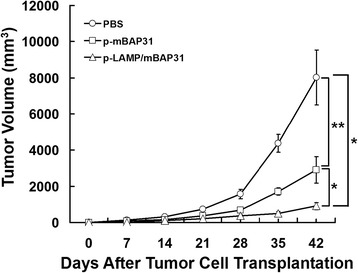
Suppression of tumor growth following mBAP31 DNA vaccine vaccinations in B16 melanoma-bearing C57BL/6 mice. C57BL/6 mice were transplanted subcutaneously with 5 × 10^4^ B16 cells per mouse at day 0, and were immunized with p-mBAP31, p-LAMP/mBAP31 or PBS on days 3, 10, 17 and 24. Tumor size was serially measured and mice were sacrificed on day 42. Student *t* test was done based on the data of day 42. *, *P* < 0.05 for the comparison of the tumor volume between the p-LAMP/mBAP31 vaccinated mice and those vaccinated with p-mBAP31 or PBS. **, *P* < 0.05 for the comparison between the p-mBAP31 vaccinated mice and those vaccinated with PBS.

The ability of therapeutic anti-tumor effects in tumor-bearing mice of mBAP31 DNA vaccine was investigated using mBAP31-positive B16 melanoma cells in C57BL/6 mice. C57BL/6 mice were transplanted subcutaneously with 5 × 10^4^ B16 cells per mouse at day 0, and were immunized with p-mBAP31, p-LAMP/mBAP31 or PBS on days 3, 10, 17 and 24. Tumor size was measured every week from day 0 and all mice were sacrificed on day 42. As shown in Figure 6, vaccination with p-mBAP31 and p-LAMP/mBAP31 could significantly inhibited the B16 tumor growth compared with PBS group (*P* < 0.05). Moreover, p-LAMP/mBAP31 vaccine had much better anti-tumor activity than that of p-mBAP31 (*P* < 0.05). These data suggested that the LAMP/mBAP31 vaccine has potent anti-tumor activity even in the established tumor. Furthermore, tumor-bearing mice treated with p-LAMP/mBAP31 vaccine resulting in increased infiltration of CD8^+^ T cells into MM tissues (Additional file 4: Figure S4).

## Disscussion

For the first time, we found that protein expression of hBAP31 were dramatically upregulated in human MM tissues when compared with human normal tissues (lung, colon, rectum, liver, ovary, breast, cervix and esophagus), with a total positive rate of 86.5% (122 of 141 cases). And predominant protein expression of mBAP31 was found in mouse B16 melanoma cells but not in mouse important organs including brain, heart, colon, lung, liver, pancreas and kidney. Northern blotting analyses have revealed that BAP31 RNA transcripts are ubiquitously expressed, but prominent BAP31 protein expression analyzed by immunohistochemistry in our study and previous reports is restricted to a minority of cells in normal human tissues although its RNA transcripts are ubiquitously expressed [13,14]. The mechanism of the discrepancy of the expression level between the mRNA and protein of BAP31 should be investigated more intensely.

For a tumor antigen to be a promising immunotherapeutic target, the following properties would be crucial: 1) the protein expression should be documented by IHC analysis, and 2) the gene should have a reasonable frequency of expression, ideally in at least 10-20% of at least one or two tumor types [19]. Using these two criteria, we have evaluated the BAP31, a gene product encoded by a gene located on Xq26, was hypothesized in the present study as a promisingly attractive vaccine target for human MMs.

The past five decades have witnessed a steady increase in the incidence of MMs [20]. While early detection, appropriate surgery and, in some cases, adjuvant therapy have improved outcomes, at least one third of patients with early-stage melanoma will develop metastases. The prognosis for patients with metastatic melanoma remains dismal. Studies have shown that biochemotherapy (chemotherapy combined with interleukin-2 and interferon) was not associated with a statistically significant survival benefit in any of the individual trials or in a pooled analysis [21-26]. The emerging field of cancer immunotherapy offers a number of exciting promising treatments and cast new light on the therapeutic strategies of the disease [5,27-32].

Currently, cancer vaccine therapy for MMs has a 2-fold focus. On the one hand, advances have been aimed at improving the effectiveness of MM vaccines based on the identification of various MM associated antigens (i.e., MAGE, GAGE, SPANXC, SSX, etc.) [33-40]. On the other hand, the immune system also inadvertently plays a supportive role in promoting the development and progression of tumors [21,41]. Because tumor cells have multiple mechanisms to escape the cytotoxicity of the immune system, polyvalent vaccines are desirable, prompting searches for novel MM associated antigens.

BAP31 is an endoplasmic reticulum protein-sorting factor that associates with newly synthesized integral membrane proteins and controls their fate (i.e., egress, retention, survival, or degradation). For the first time, we identified high protein expression level of hBAP31 in human MM tissues, with a total positive rate of 86.5%. These results suggested that hBAP31 might be a promisingly attractive vaccine target for human MMs.. Moreover, our preliminary work suggested that depletion of BAP31 has a negative impact on the proliferation of tumor cells, indicating that BAP31 may play an important role in tumorigenesis. Further experiments showed that BAP31 might facilitate the resistance to apoptosis triggered by multiple chemotherapeutic drugs including cisplatin, VP-16 and taxol *in vitro*, which was used for the adjuvant chemotherapy various cancers (unpublished data).

To enhance the immune response against mBAP31 DNA vaccines, we developed a LAMP-mBAP31 chimera DNA vaccine based on previous reports on its function of enhancing T cell responses against various virus and tumor antigens. When C57BL/6 mice were immunized with LAMP-mBAP31 chimera DNA vaccine, stronger cellular response to mBAP31, CTL cytotoxicity and anti-tumor effects in the therapeutic model were observed than mice vaccinated with p-mBAP31 DNA vaccine. Moreover, no autoimmune disorders were observed in the vaccinated mice, suggesting the safety of vaccination with mBAP31 DNA vaccine. These are the first data supporting a vaccine targeting BAP31 that will be applicable to human MMs.

In conclusion, we demonstrated that immunization with a novel LAMP-mBAP31 chimera DNA vaccine can successfully generate mBAP31-specific anti-tumor immunity in mice supports further evaluation regarding the promising clinical efficacy of this vaccine and the preclinical trials is ongoing in our lab.

## Additional files

Additional file 1: Figure S1. Expression of mBAP31 protein in normal mouse tissues detected by Western blot. Western blot analysis was performed to determine the expression profiles of mBAP31 protein in colon (Lane 1), lung (Lane 2), liver (Lane 3), spleen (Lane 4), skin (Lane 5) and kidney (Lane 6). There are no significant 28KD bands in the film, suggesting low protein expression of mBAP31 in mouse normal tissues mentioned above. Anti-β-actin mAb was used as internal standard for all samples. Additional file 2: Figure S2. Expression of mBAP31 protein in B16 cells, CT26 cells and siRNA B16 cells detected by Western blot. Western blot analysis was performed to determine the expression profiles of mBAP31 protein in B16 cells (Lane 1), 1H12 cells (Lane 2, pSUPER-Control-transfected clonal cell line,), 2E8 cells (Lane 3, pSUPER-mBAP31-transfected clonal cell line) and CT26 cells (Lane 4). There is a special 28KD band in the film suggesting high protein expression of mBAP31 in B16 cells, 1H12 cells and CT26 cells. Expression of mBAP31 was significantly down-regulated in 2E8 cells. Anti-β-actin mAb was used as internal standard for all samples. Additional file 3: Figure S3. Expression of mBAP31 protein in mBAP31 DNA vaccine transfected cells. No significant difference in the fluorescence intensity of the levels of EGFP-tagged mBAP31 (A) and LAMP/mBAP31 (B) expression in transfected 293T cells when analyzed by fluorescence microscopy. Additional file 4: Figure S4. Increased infiltration CD8^+^ T cells into MM tissues of tumor-bearing mice treated with p-LAMP/mBAP31. To obtain tumor infiltrated lymphocytes (TIL), tumor grafts were cut into small pieces and digested with collagenase and DNase I followed by Ficoll-Hypaque separation. Then the TILs were incubated with FITC-labeled anti-CD8 mAb and analyzed by flow cytometry. The infiltration levels of TILs in tumor-bearing animals treated with p-LAMP/mBAP31 (C) showed a higher degree of CD8^+^ T cell infiltration into the tumor tissues than animals treated with p-mBAP31 (B) or PBS (A).

## References

[1] DeSantis CE, Lin CC, Mariotto AB, Siegel RL, Stein KD, Kramer JL (2014). Cancer treatment and survivorship statistics, 2014. CA Cancer J Clin.

[2] Kimbrough CW, McMasters KM, Davis EG (2014). Principles of surgical treatment of malignant melanoma. Surg Clin North Am.

[3] Vogt T (2014). Therapy of metastatic malignant melanoma: on the way to individualized disease control. Adv Exp Med Biol.

[4] Shimanovsky A, Jethava A, Dasanu CA (2013). Immune alterations in malignant melanoma and current immunotherapy concepts. Expert Opin Biol Ther.

[5] Zarour HM, Ferrone S (2011). Cancer immunotherapy: progress and challenges in the clinical setting. Eur J Immunol.

[6] Vici P, Mariani L, Pizzuti L, Sergi D, Di Lauro L, Vizza E (2014). Immunologic treatments for precancerous lesions and uterine cervical cancer. J Exp Clin Cancer Res.

[7] Nicholaou T, Ebert LM, Davis ID, McArthur GA, Jackson H, Dimopoulos N (2009). Regulatory T-cell-mediated attenuation of T-cell responses to the NY-ESO-1 ISCOMATRIX vaccine in patients with advanced malignant melanoma. Clin Cancer Res.

[8] Curioni-Fontecedro A, Nuber N, Mihic-Probst D, Seifert B, Soldini D, Dummer R (2011). Expression of MAGE-C1/CT7 and MAGE-C2/CT10 predicts lymph node metastasis in melanoma patients. PLoS One.

[9] Wang B, Heath-Engel H, Zhang D, Nguyen N, Thomas DY, Hanrahan JW (2008). BAP31 interacts with Sec61 translocons and promotes retrotranslocation of CFTRDeltaF508 via the derlin-1 complex. Cell.

[10] Breckenridge DG, Nguyen M, Kuppig S, Reth M, Shore GC (2002). The procaspase-8 isoform, procaspase-8 L, recruited to the BAP31 complex at the endoplasmic reticulum. Proc Natl Acad Sci U S A.

[11] Ng FW, Nguyen M, Kwan T, Branton PE, Nicholson DW, Cromlish JA (1997). p28 Bap31, a Bcl-2/Bcl-XL- and procaspase-8-associated protein in the endoplasmic reticulum. J Cell Biol.

[12] Ng FW, Shore GC (1998). Bcl-XL cooperatively associates with the Bap31 complex in the endoplasmic reticulum, dependent on procaspase-8 and Ced-4 adaptor. J Biol Chem.

[13] Adachi T, Schamel WW, Kim KM, Watanabe T, Becker B, Nielsen PJ (1996). The specificity of association of the IgD molecule with the accessory proteins BAP31/BAP29 lies in the IgD transmembrane sequence. EMBO J.

[14] Manley HA, Lennon VA (2001). Endoplasmic reticulum membrane-sorting protein of lymphocytes (BAP31) is highly expressed in neurons and discrete endocrine cells. J Histochem Cytochem.

[15] Goldoni AL, Maciel M, Rigato PO, Piubelli O, de Brito CA, Melo A (2011). Mucosal and systemic anti-GAG immunity induced by neonatal immunization with HIV LAMP/gag DNA vaccine in mice. Immunobiology.

[16] Yang K, Sun K, Srinivasan KN, Salmon J, Marques ET, Xu J (2009). Immune responses to T-cell epitopes of SARS CoV-N protein are enhanced by N immunization with a chimera of lysosome-associated membrane protein. Gene Ther.

[17] Osada T, Woo CY, McKinney M, Yang XY, Lei G, Labreche HG (2009). Induction of Wilms' tumor protein (WT1)-specific antitumor immunity using a truncated WT1-expressing adenovirus vaccine. Clin Cancer Res.

[18] Song C, Wang F, Xu Z, Hu J, Tao H, Yang A (2009). Monoclonal antibodies against human BAP31 for immunocytochemistry. Hybridoma (Larchmt).

[19] Simpson AJ, Caballero OL, Jungbluth A, Chen YT, Old LJ (2005). Cancer/testis antigens, gametogenesis and cancer. Nat Rev Cancer.

[20] Edwards BK, Brown ML, Wingo PA, Howe HL, Ward E, Ries LA (2005). Annual report to the nation on the status of cancer, 1975–2002, featuring population-based trends in cancer treatment. J Natl Cancer Inst.

[21] Hamm C, Verma S, Petrella T, Bak K, Charette M (2008). Biochemotherapy for the treatment of metastatic malignant melanoma: a systematic review. Cancer Treat Rev.

[22] Middleton MR, Grob JJ, Aaronson N, Fierlbeck G, Tilgen W, Seiter S (2000). Randomized phase III study of temozolomide versus dacarbazine in the treatment of patients with advanced metastatic malignant melanoma. J Clin Oncol.

[23] Eton O (2005). Chemotherapy, cytokines, and biochemotherapy for melanoma. Cancer Chemother Biol Response Modif.

[24] Petrella T, Quirt I, Verma S, Haynes AE, Charette M, Bak K (2007). Single-agent interleukin-2 in the treatment of metastatic melanoma: a systematic review. Cancer Treat Rev.

[25] Eton O, Legha SS, Bedikian AY, Lee JJ, Buzaid AC, Hodges C (2002). Sequential biochemotherapy versus chemotherapy for metastatic melanoma: results from a phase III randomized trial. J Clin Oncol.

[26] Lewis KD, Robinson WA, McCarter M, Pearlman N, O'Day SJ, Anderson C (2006). Phase II multicenter study of neoadjuvant biochemotherapy for patients with stage III malignant melanoma. J Clin Oncol.

[27] Tiwari M (2010). From tumor immunology to cancer immunotherapy: miles to go. J Cancer Res Ther.

[28] Pandolfi F, Cianci R, Lolli S, Dunn IS, Newton EE, Haggerty TJ (2008). Strategies to overcome obstacles to successful immunotherapy of melanoma. Int J Immunopathol Pharmacol.

[29] Grange JM, Krone B, Stanford JL (2009). Immunotherapy for malignant melanoma–tracing Ariadne's thread through the labyrinth. Eur J Cancer.

[30] Mayayo SL, Prestigio S, Maniscalco L, Rosa GL, Arico A, Maria RD (2011). Chondroitin sulfate proteoglycan-4: a biomarker and a potential immunotherapeutic target for canine malignant melanoma. Vet J.

[31] Chiarion Sileni V, Pigozzo J, Ascierto PA, Grimaldi AM, Maio M, Di Guardo L (2014). Efficacy and safety of ipilimumab in elderly patients with pretreated advanced melanoma treated at Italian centres through the expanded access programme. J Exp Clin Cancer Res.

[32] Altomonte M, Di Giacomo A, Queirolo P, Ascierto P, Spagnolo F, Bajetta E (2013). Clinical experience with ipilimumab 10 mg/kg in patients with melanoma treated at Italian centres as part of a European expanded access programme. J Exp Clin Cancer Res.

[33] van der Bruggen P, Traversari C, Chomez P, Lurquin C, De Plaen E, Van den Eynde B (1991). A gene encoding an antigen recognized by cytolytic T lymphocytes on a human melanoma. Science.

[34] Van den Eynde B, Peeters O, De Backer O, Gaugler B, Lucas S, Boon T (1995). A new family of genes coding for an antigen recognized by autologous cytolytic T lymphocytes on a human melanoma. J Exp Med.

[35] Muscatelli F, Walker AP, De Plaen E, Stafford AN, Monaco AP (1995). Isolation and characterization of a MAGE gene family in the Xp21.3 region. Proc Natl Acad Sci U S A.

[36] Kirkin AF, Dzhandzhugazyan KN, Zeuthen J (2002). Cancer/testis antigens: structural and immunobiological properties. Cancer Invest.

[37] Pold M, Zhou J, Chen GL, Hall JM, Vescio RA, Berenson JR (1999). Identification of a new, unorthodox member of the MAGE gene family. Genomics.

[38] Van den Eynde B, Brichard VG (1995). New tumor antigens recognized by T cells. Curr Opin Immunol.

[39] Zendman AJ, Cornelissen IM, Weidle UH, Ruiter DJ, van Muijen GN (1999). CTp11, a novel member of the family of human cancer/testis antigens. Cancer Res.

[40] Tureci O, Sahin U, Schobert I, Koslowski M, Scmitt H, Schild HJ (1996). The SSX-2 gene, which is involved in the t(X;18) translocation of synovial sarcomas, codes for the human tumor antigen HOM-MEL-40. Cancer Res.

[41] Ralph SJ (2007). An update on malignant melanoma vaccine research: insights into mechanisms for improving the design and potency of melanoma therapeutic vaccines. Am J Clin Dermatol.

