# Supplementary figures and images for: BAP31, a promising target for the immunotherapy of malignant melanomas
Source: J Exp Clin Cancer Res. 2015 Apr 18;34(1):36. doi: 10.1186/s13046-015-0153-6 (PMC4405826; doi:10.1186/s13046-015-0153-6)

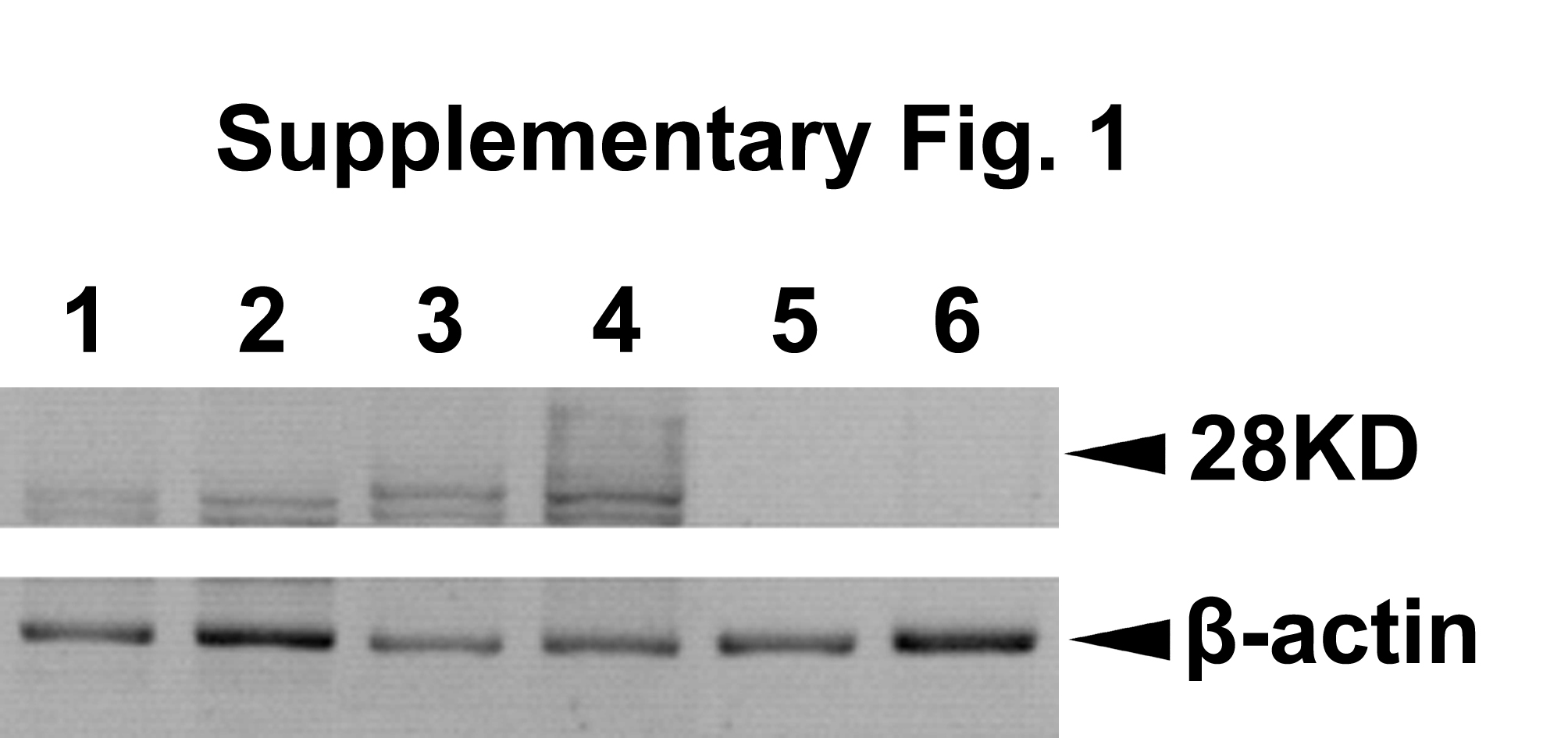

Supplement: Additional file 1: Figure S1. — Expression of mBAP31 protein in normal mouse tissues detected by Western blot. Western blot analysis was performed to determine the expression profiles of mBAP31 protein in colon (Lane 1), lung (Lane 2), liver (Lane 3), spleen (Lane 4), skin (Lane 5) and kidney (Lane 6). There are no significant 28KD bands in the film, suggesting low protein expression of mBAP31 in mouse normal tissues mentioned above. Anti-β-actin mAb was used as internal standard for all samples. [file 13046_2015_153_MOESM1_ESM.tiff]

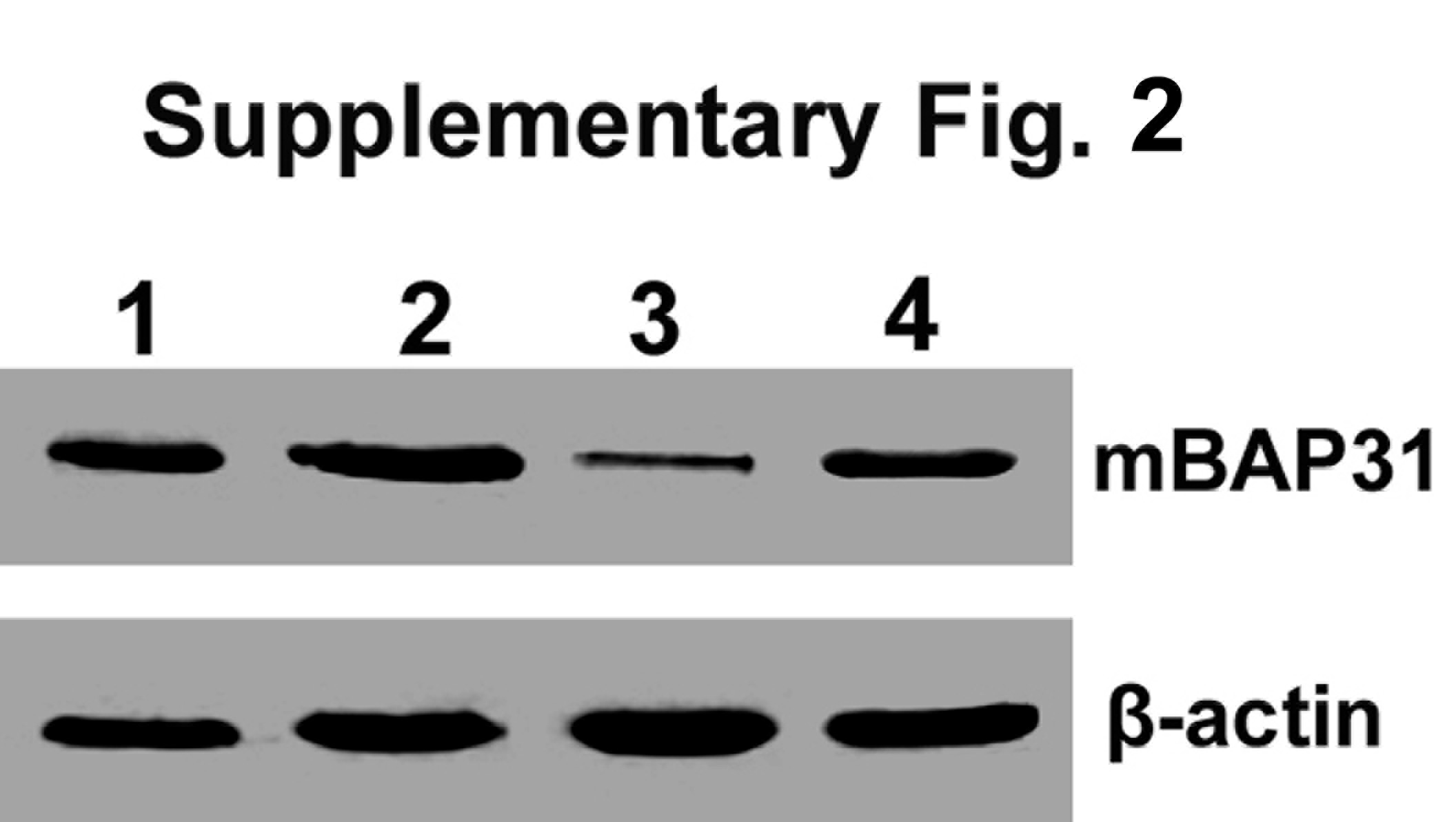

Supplement: Additional file 2: Figure S2. — Expression of mBAP31 protein in B16 cells, CT26 cells and siRNA B16 cells detected by Western blot. Western blot analysis was performed to determine the expression profiles of mBAP31 protein in B16 cells (Lane 1), 1H12 cells (Lane 2, pSUPER-Control-transfected clonal cell line,), 2E8 cells (Lane 3, pSUPER-mBAP31-transfected clonal cell line) and CT26 cells (Lane 4). There is a special 28KD band in the film suggesting high protein expression of mBAP31 in B16 cells, 1H12 cells and CT26 cells. Expression of mBAP31 was significantly down-regulated in 2E8 cells. Anti-β-actin mAb was used as internal standard for all samples. [file 13046_2015_153_MOESM2_ESM.tiff]

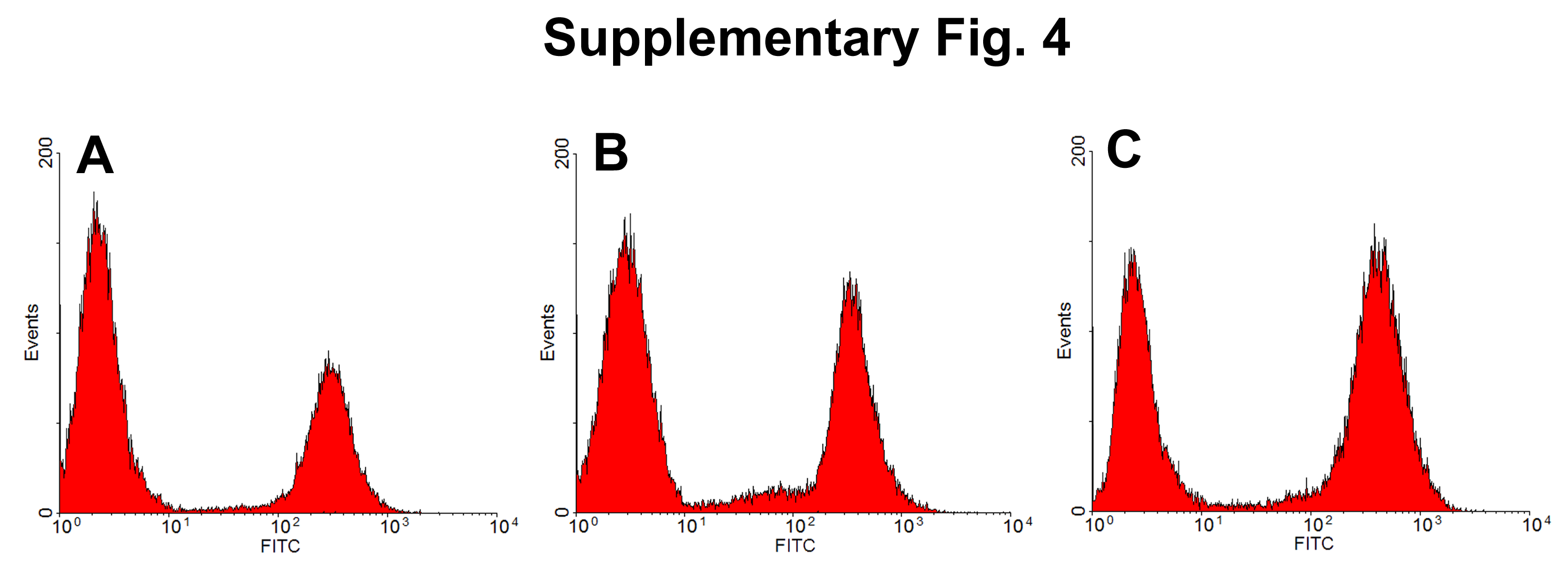

Supplement: Additional file 4: Figure S4. — Increased infiltration CD8+ T cells into MM tissues of tumor-bearing mice treated with p-LAMP/mBAP31. To obtain tumor infiltrated lymphocytes (TIL), tumor grafts were cut into small pieces and digested with collagenase and DNase I followed by Ficoll-Hypaque separation. Then the TILs were incubated with FITC-labeled anti-CD8 mAb and analyzed by flow cytometry. The infiltration levels of TILs in tumor-bearing animals treated with p-LAMP/mBAP31 (C) showed a higher degree of CD8+ T cell infiltration into the tumor tissues than animals treated with p-mBAP31 (B) or PBS (A). [file 13046_2015_153_MOESM4_ESM.tiff]
